# Clinical Impact of a Next-Generation Sequencing Approach for Glioblastoma Patients

**DOI:** 10.3390/cancers17050744

**Published:** 2025-02-22

**Authors:** Catalina Vivancos Sánchez, María Isabel Esteban Rodríguez, Alberto Peláez García, Mario Taravilla-Loma, Víctor Rodríguez-Domínguez, Carlos Rodríguez-Antolín, Rocío Rosas-Alonso, Itsaso Losantos-García, Alberto Isla Guerrero, María Luisa Gandía-González

**Affiliations:** 1 Neurosurgery Department, La Paz University Hospital, Paseo de la Castellana 261, 28046 Madrid, Spain; mario.taravilla@salud.madrid.org (M.T.-L.); victor.rodriguez@salud.madrid.org (V.R.-D.); alberto.isla@salud.madrid.org (A.I.G.); marialuisa.gandia@salud.madrid.org (M.L.G.-G.); 2 Pathology Department, La Paz University Hospital, Paseo de la Castellana 261, 28046 Madrid, Spain; misabel.esteban@salud.madrid.org; 3 Cancer Epigenetics Laboratory, INGEMM, La Paz University Hospital, Biomarkers and Experimental Therapeutics in Cancer, IdiPAZ, 28046 Madrid, Spain; crodrigueza@salud.madrid.org (C.R.-A.); rocio.rosas@salud.madrid.org (R.R.-A.); 4 Molecular Pathology and Therapeutic Targets Group, La Paz University Hospital, IdiPAZ, Paseo de la Castellana 261, 28046 Madrid, Spain; alberto.pelaez@salud.madrid.com; 5 Bioinformatics Unit, INGEMM, La Paz University Hospital, Paseo de la Castellana 261, 28046 Madrid, Spain; 6 Biostatistics Department, La Paz University Hospital, IdiPAZ, Paseo de la Castellana 261, 28046 Madrid, Spain; itsaso.losantos@salud.madrid.org

**Keywords:** glioblastoma, high-grade gliomas, next-generation sequencing, genomic alterations, targeted molecular therapy, clinical trials

## Abstract

**Objectives:** The purpose of this study is to assess the clinical impact of next-generation sequencing (NGS), as an increasingly available and advantageous tool, for glioblastoma patients. **Methods:** Adult patients aged less than 65, and surgically treated for glioblastoma between 2010–2021, were included. Tumor samples were analyzed with NGS using the Oncomine Comprehensive v3 (OCA) panel and Ion Reporter Genexus v5.9.1 (Thermo Fisher Scientific). **Results:** Thirty-two patients were included, with a median age of 47.7 years and a median overall survival of 25 months. Identification of mutations by NGS resulted in a change in diagnosis in two cases. In all patients but one, at least one genetic alteration was detected (median of three per patient), most commonly EGFR amplification. In 93.7% of patients, biomarkers that make them potentially eligible for a clinical trial were found. No survival differences were seen regarding genetic alterations, although a trend towards better survival for those patients without CDK4 mutation was observed (*p* = 0.088). **Conclusions:** The use of NGS provides useful information for diagnosis, especially in young patients, and it will probably become valuable for clinical decision-making as more therapeutic targets and treatments emerge. For the moment, it is crucial for scientific progress to happen.

## Simple Summary

High-grade gliomas, especially glioblastoma, persist in having a highly unfavourable prognosis despite recent advances and maximal surgical removal. Multiple genomic alterations have recently been described as diagnostic markers, prognostic factors, and targets for molecular therapy. Our retrospective study aimed to evaluate the clinical impact of next-generation sequencing (NGS) as a tool to assess tumoral genetic alterations in patients affected by glioblastoma. We confirmed its usefulness in a population of 32 surgically treated, young (<65 years old) glioblastoma patients: identification of mutations resulted in a change in diagnosis in two cases; in all patients but one, at least one genetic alteration was detected; and in 93.7% of patients, biomarkers that make them potentially eligible for a clinical trial were found. NGS is then useful not only for diagnostics but also for the detection of potential experimental targets.

## 1. Introduction

High-grade gliomas (HGG) remain central nervous system (CNS) tumors with a very poor survival rate, despite recent advances and maximal surgical removal. Molecular advances have led to a more accurate classification of HGG in CNS5 World Health Organization (WHO) 2021 [1,2]: for glioblastoma (GB) diagnosis, a characteristic histological appearance (poorly differentiated astrocytes, with nuclear atypia, prominent microvascular proliferation, and/or necrosis) combined with an isocitrate dehydrogenase (IDH) wildtype genotype is sufficient. In the absence of a histological GB phenotype, IDH wildtype tumors that harbor EGFR (epidermal growth factor receptor) amplification, TERT (telomerase reverse transcriptase) promoter mutation, or chromosome 7 gains and 10 losses (genomic findings correlated with poor clinical outcome and prognosis) can lead to the diagnosis of GB [2,3]. However, reaching the precise diagnosis sometimes presents a problem in terms of time and required techniques. Traditional detection of mutations, intragenic deletions, and gene fusions requires time-consuming methods, such as Sanger or pyro sequencing on DNA or cDNA, fluorescence in situ hybridization, multiplex-ligation assays, and others [4].

On the other hand, HGG’s resistant nature to available treatments poses a therapeutic problem [3,5]. When focusing on glioblastoma patients, even following the same treatment regimens, very distant outcomes are observed. Lately, various research teams have delineated the genomic landscape of gliomas, uncovering a complex genomic landscape marked by numerous molecular changes, some of which are recognizable targets for biological treatments [3]. Next-generation sequencing (NGS) has not only shown to be able to refine but even independently identify an integrated diagnosis of brain tumors with pathognomonic genetic alterations [4]. Moreover, it can assess an extensive set of genomic targets with high precision and sensitivity due to high sequencing coverage, and it is proven to result in a five-workday workflow [4]. However, most genomic alterations seen in GB lack, nowadays, effective therapeutic agents (EGFR-amplification, EGFRVIII-mutation, TERT-promotor mutation) [5]. A different picture can be seen in pediatric neuro-oncology, where the use of NGS is already recommended in clinical routine as BRAF inhibitors can be used for the treatment of BRAF-mutant higher-grade gliomas, alone or in combination with mitogen-activated protein kinase (MEK) inhibitors [5]. Also, the identification of NTRK (neurotrophic tyrosine receptor kinase) fusions in a rare subset of pediatric and adult gliomas focused attention on pan-oncologic NTRK inhibitors [5,6].

Finally, it is important to note that even though maximal safe surgical resection is the current most important prognostic factor for survival in GB patients, a small subset of patients (those who progress previous to or under initial concomitant adjuvant treatment) do not take good advantage of resective surgery in terms of survival [7,8]. Given that GB associates with a burden of neurological morbidity itself, and sometimes by surgery, quality of life and surgery implications should be taken into account, especially for this group of patients. Consequently, assessment of differential tumor characteristics within these patients could lead to the development of more effective and tolerable treatment strategies for glioblastoma.

Since NGS technologies are increasingly available, they are nowadays commonly used in European neuro-oncology primary care centers in clinical routine, although they have not been established yet as diagnostic tools for brain tumors in the adult population because of seldom changing the course of treatment to date [5]. NGS does not have high-level evidence of clinical impact, but as NGS might detect targetable mutations, providing information about potentially actionable gene aberrations, it has growing attention in research and clinical practice, especially when used in younger glioblastoma patients [5].

With the recent recognition of genetic markers as prognostic factors and treatment targets in neuro-oncology, the increasing availability and advantages of NGS (not yet officially recommended in clinical routine for adult patients), and the diagnostic and therapeutic problems previously defined, assessing the actual clinical impact of a next-generation sequencing approach for glioblastoma patients is the aim of our study.

## 2. Materials and Methods

### 2.1. Study Design

A total of 32 retrospective cases were assessed. Selection criteria were as follows: adult patients (≥18 y.o.), who had been surgically treated (resective surgery or biopsy) at our institution between 2010–2021, with an informed diagnosis of glioblastoma; IDH not mutated; grade 4—according to current WHO classification criteria at the time of diagnosis, and consistent with WHO 2021 criteria based on available information; minimum follow-up of 1 year; and early postoperative brain magnetic resonance imaging (MRI, <72 h from surgery) availability. Only younger patients (under 65 years of age) were included to homogenize the sample, to avoid the effect of age on survival, and since these are the patients most likely to undergo an NGS study in current clinical practice. Samples were obtained from the Pathology Department of La Paz University Hospital (Madrid). Information on demographic, baseline, and follow-up characteristics was collected retrospectively from the patients’ medical records. Progression was determined based on clinical evaluation and modified Response Assessment in Neuro-Oncology (RANO) criteria from MRI [9]. This study was approved by the local Ethics Committee.

### 2.2. Thermo Fisher’s Oncomine Comprehensive v3 (OCA)—Genexus

All samples contained a minimum of 20% tumor cell-derived nuclei, as determined by histopathological evaluation. Ten-micrometer sections of Formalin-Fixed Paraffin-Embedded (FFPE) samples were deparaffinized in xylene and hydrated by graded alcohols to water. Samples were washed in phosphate-buffered saline and digested in proteinase K overnight at 56 °C. Nucleic acid extraction was performed using the Ion Torrent Genexus purification system (Thermo Fisher Scientific, Waltham, MA, USA). The amount of input DNA and RNA ranged from 10–20 ng (the manufacturer’s recommended input is 20 ng).

The OCav3 is a targeted amplicon-based assay that can detect 161 unique genes. OCA v3 (Thermo Fisher Scientific) is an expanded targeted amplicon panel designed for solid tumors that detects relevant single nucleotide variants, amplifications, gene fusions, and indels for 161 key cancer driver genes. The full list of genes included in this panel is available online (https://www.thermofisher.com/order/catalog/product/A35805 (accessed on 21 February 2025)). Genomic data were analyzed and alterations were detected using Ion Reporter Genexus v5.9.1 Oncomine Reporter version 2024.007 (005) data software (Thermo Fisher Scientific, Waltham, MA, USA). The variant call format file and the integrated genomic viewer were also manually reviewed. Only variants in coding regions, promoter regions, or splice variants were retained. All genes subject to copy number analysis were sorted by their chromosomal loci and the average copy number of each gene was documented.

### 2.3. Immunohistochemistry and Mutation Testing of pTERT

Immunohistochemistry was performed on 4 μm sections by the Envision method (Dako-Agilent, Glostrup, Denmark) in an automated Omnis platform (Dako-Agilent) according to the manufacturer’s instructions with the following antibodies: IDH1-R132H (dilution 1:20, Clone H09, Dianova, Hamburg, Germany) and ATRX (dilution 1:100; Clone AX1; Dianova, Hamburg, Germany) Supplementary Figure S1. The nuclei were counterstained with hematoxylin.

Selected FFPE blocks containing > 20% of viable tumor tissue were used to extract DNA by QIAamp FFPE tissue kit (Qiagen, Hilden, Germany) and used for polymerase chain reaction (PCR) and Sanger sequencing. Two hotspot mutations in the TERT core promoter, C228T and C250T, correspond to positions 124 and 146-bp upstream of the ATG site (Supplementary Figure S2). PCR was performed in a 20 μL reaction with these specific primers (5′-3′): CAGCGCTGCCTGAAACTC and GTCCTGCCCCTTCACCTT. PCR products were subjected to electrophoresis on 2% agarose gels and bidirectional sequencing was performed using a BigDye Terminator v1.1 Cycle Sequencing Kit (Applied Biosystems, Foster City, CA, USA) on an ABI 3130XL Genetic Analyzer (Applied Biosystems). The results were regarded as mutation-positive if a mutation was detected in both forward and reverse read directions.

### 2.4. Imaging

Surgical resection was defined based on the extent of resection (EOR) calculated by volumetry of contrast enhancement in early postoperative 3 Tesla magnetic resonance imaging compared to preoperative MRI: EOR < 50% biopsy, EOR 50–90% partial resection, EOR 90–99% subtotal resection, and EOR >99% gross total resection (Supplementary Figure S3). MRI was performed in a 3 Tesla magnet (MAGNETOM Skyra, Siemens, Erlangen, Germany). It included sagittal 3D-T1w-SPACE (sampling perfection with application-optimized contrast, TR: 650 ms, TE: 11 ms, and 1 mm isotropic resolution) before and after gadolinium (Dotarem R 0.2 mL/kg IV bolus infused at a rate of 2 mL/s) administration.

### 2.5. Statistical Analysis

Qualitative data were presented as absolute frequencies and percentages, while quantitative data were reported as medians and interquartile ranges. The survival study utilized Kaplan–Meier analysis alongside log-rank tests to compare survival functions, in addition to Cox regression. The latter was also used to investigate the risk of variables that were significantly associated with survival.

All statistical tests were considered bilateral and *p*-values of less than 0.05 were considered significant. Data were analyzed with the statistical program R version 4.3.3 (R Core Team, 2024).

## 3. Results

### 3.1. Patient Characteristics

Thirty-two patients were included in the analysis and their tumor samples were profiled with the Oncomine^TM^ gene panel. Demographic and relevant clinical–pathological characteristics are shown in Table 1. The median age was 47.7 years, and the majority of patients were men (68.8%). Headache (50%) and seizures (37.5%) were the most frequent form of presentation. Baseline functional status was ECOG (Eastern Cooperative Oncology Group performance status) 0 for 65.6% of patients, and ECOG 1 for the rest. Samples submitted for NGS were obtained from the primary tumor for all patients. Thirteen (40.6%) patients had surgery at progression. Mean overall survival was 32 (SD 3.61) months. Median overall survival was 25 months [IC95% (18, 39)]. Mean progression-free survival was 18,6 months (SD 2.62). Median progression-free survival was 13 months [IC95% (10, 23)]. 30 patients were dead at the moment of data analysis; two were alive with a follow-up and survival of 55 and 67 months, respectively. Tumor volumetry data are described in Table 2.

### 3.2. Survival Predictive Factors

In the present patient cohort, no survival differences were seen regarding preoperative tumor volumetry (*p* = 0.25) nor the extent of resection (EOR, *p* = 0.32). Age did not affect survival as well (*p* = 0.736). Postoperative contrast-enhancing tumor volumetry showed a trend towards longer survival for smaller postoperative volumes (*p* = 0.0551). A trend towards better survival for those patients with MGMT promoter methylation was observed, although significance standards were not reached (*p* = 0.0516, HR = 0.778 [IC95%= (0.365, 1.659)]) (Supplementary Figure S4).

**Figure 1:**
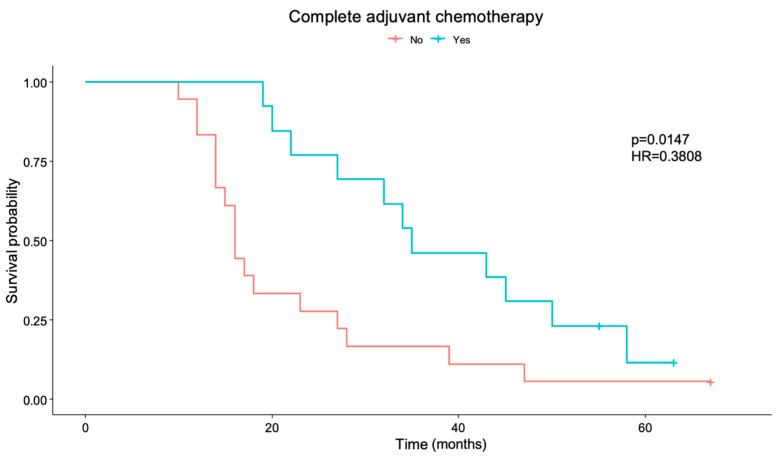
Overall Survival for completed planned adjuvant temozolomide chemotherapy (*p* = 0.014). HR: Hazard ratio. Number of patients who complete adjuvant chemotherapy: “no” = 19, “yes” = 13.

Survival differences exist for patients who completed planned adjuvant temozolomide chemotherapy (*p* = 0.014), with a Hazard ratio (HR) of 0.3808 [IC95%= (0.1754, 0.8269)], as shown in Figure 1. Those patients who completed chemotherapy have a 61.92% reduced exitus risk compared with those who did not complete the planned adjuvant chemotherapy treatment.

**Figure 2:**
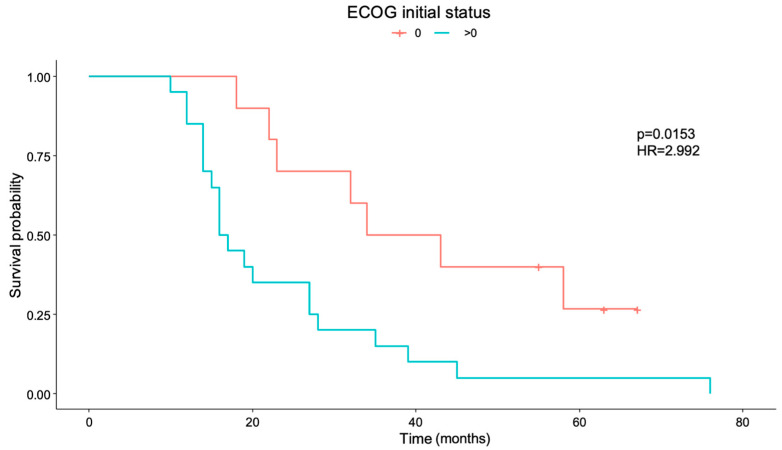
Overall survival for ECOG scale—Eastern Cooperative Oncology Group (*p* = 0.015). HR: hazard ratio. Number of patients who present ECOG initial status: “0” = 21, “>0” = 11.

Survival differences also exist for patients with initial ECOG (Eastern Cooperative Oncology Group scale) 0 compared to patients with worse functional status at diagnosis (ECOG >0), *p* = 0.015 [HR = 2.992 IC95% = (1.234, 7.255)], as shown in Figure 2.

### 3.3. Next-Generation Sequencing Quality Control and Variant Analysis

The alterations detected by the OCAv3 are listed in Table 3. The default quality control (QC) parameters established by the instrument were used, including median absolute values of all pairwise differences (MAPD) between 0 and 0.4 and the detection of at least five out of seven internal RNA control targets. Variants within exonic regions or splice site regions (variants located within the first 3 nucleotides of the 50 or 30 end), classified as SNVs (single nucleotide variants) or indels, were annotated. In addition, the coverage criteria and Phred quality score were based on the cut-off points used in our current clinical setting. On the other hand, since the Ion Torrent platform has a known limitation in homopolymeric regions (TERT promoter), showing a lower accuracy when reading lengths longer than 5 bp of homonucleotides, we performed sequencing of this region to obtain higher confidence in the results obtained. Additionally, we inferred possible germline variants based on the databases ‘1000 Genomes’ and ‘GnomAD/ExAc’. Variants classified as benign or probably benign germline variants and reviewed by expert panels in ClinVar were excluded from the identified variants.

### 3.4. Genetic Alterations

The identification of mutations by NGS would result nowadays in a change in diagnosis in two cases (6.25% of the patients): the first one, an astrocytoma, IDH mutated, grade 4, in a 34-year-old patient with IDH mutation at p.R132G (not previously identified by immunohistochemistry that targets R132H); and the second one, an H3G34 mutated Diffuse Hemispheric Glioma in a 28-year-old patient, with ATRX (alpha-thalassemia/mental retardation, X-linked) and H3G34 mutations.

**Figure 3:**
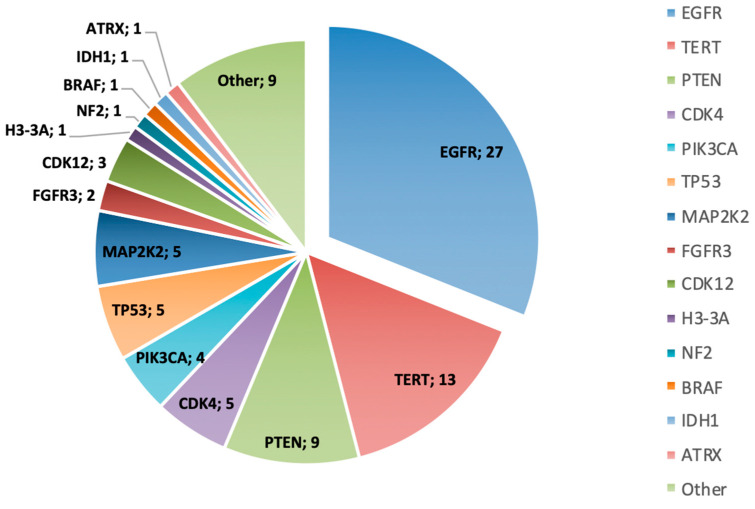
Distribution of altered genes. Numbers reflect the number of patients with an alteration of each gene. Other: PMS2, PTPRZ1-MET.P1M2 fusion, KRAS, ATM, PDGFRA, and MYCN.

In all patients but one, at least one genetic alteration (GA) was detected, most commonly EGFR amplification (13 patients) and TERT alterations (13 patients), followed by PTEN (Phosphatase and Tensin Homolog deleted on Chromosome 10) alterations in 9 patients. The median number of GAs identified per patient was three. Furthermore, in 30 out of 32 of the patients (93.7%), biomarkers that make them potentially eligible for a clinical trial were found. They are described in Table 3 and were distributed as shown in Figure 3.

**Figure 4:**
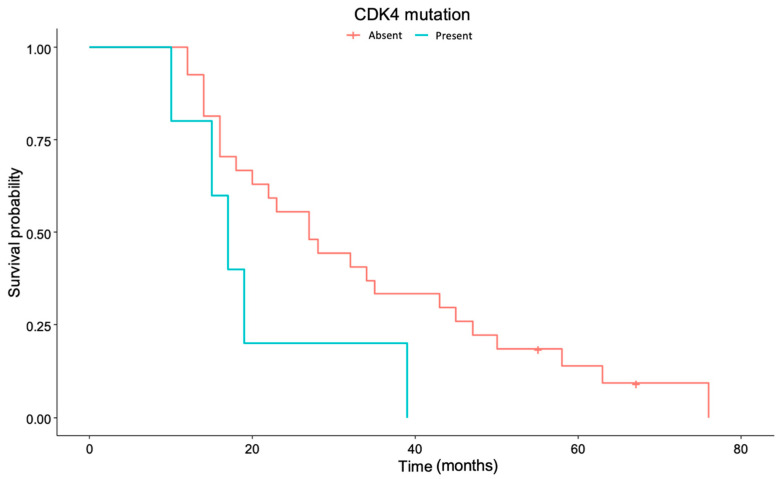
Overall survival for CDK4 (cyclin-dependent kinase 4) mutation (*p* = 0.088). Number of patients who present CDK4 mutation: “absent” = 27, “present” = 5.

In the present patient cohort, no survival differences were seen regarding EGFR amplification (*p* = 0.582) or EGFR VIII (*p* = 0.148), TERT mutation (*p* = 0.970), PIK3CA (phosphatidylinositol-4,5-bisphosphate 3-kinase catalytic subunit alpha) mutation (*p* = 0.419), or TP53 (tumor protein 53, *p* = 0.713). A trend towards better survival for those patients without CDK4 (cyclin-dependent kinase 4) mutation was observed, although significance standards were not reached (*p* = 0.088) (Figure 4).

### 3.5. Treatment Decision Impact

As NGS was retrospectively assessed, in our cohort actual treatment decision impact was not feasible. However, it is remarkable that for 30 out of 32 patients (93.7%), biomarkers that would have made them potentially eligible for a clinical essay were found. On the other hand, even if NGS detected two cases with an initial inaccurate diagnosis, those patients would have probably received the same treatment initially with the Stupp protocol given the unchanged WHO grade 4. In any case, the detection of IDH1 p.(R132G) c.394C > G mutation by NGS as observed in “patient 2” is important, as it is an example of how NGS enables the detection of IDH mutations that cannot be assessed through immunohistochemistry. Currently, targeted therapy is available for patients with low-grade gliomas harboring IDH mutations (vorasidenib) [10].

## 4. Discussion

When focusing on IDH-wt glioblastoma patients who follow the same treatment regimens, contrasting survival outcomes are observed. To date, even for recently defined CNS WHO grade 4 subtypes of diffuse gliomas other than GB, which are more prevalent in younger patients (for example, H3G34-mutant diffuse hemispheric gliomas or IDH-mutant grade 4 astrocytoma), the therapeutic strategies remain similar for all of them [5]. In glioma, it is well known that O-6-methylguanine-DNA methyltransferase (MGMT) promoter methylation status is both prognostic and predictive of treatment outcome with temozolomide, so treatment decisions in clinical routine for glioblastoma are mainly based on it.

Other than that, in the last decade, there have been several unsuccessful clinical trials for high-grade gliomas, but in other malignancies, targeted therapies have been successful [5]. Detection and utility of predictive biomarkers have shown clinical improvement and survival benefits for matched targeted therapies in several types of malignancies (BRAF in melanoma or EGFR in non-small cell lung cancer) [3]. That is why NGS technologies have attracted increasing attention.

Before talking about therapeutic options and investigation, the reality is that NGS is increasingly becoming part of the diagnostic routine in a large number of neuro-oncology centers, although no specific panel for brain tumor diagnostics has been established for the moment [4]. In a Swiss nationwide investigation on the utilization of NGS in the clinical care of GB patients, they found out that in four out of eight neuro-oncology centers, an OCA panel is used (in another one, a TS500 panel is used; in two centers, a custom-made panel is used; and in the last one, no NGS is used) [5]. In those centers, younger age (younger than 60), fitness status, and lack of suitability for standard treatment (or having exhausted standards of care) are the most relevant criteria for NGS application [5].

These criteria are similar to the ones used in our center, especially younger age, which is one of the reasons why we only included patients younger than 65 years old in our study. The second reason is that age does not affect survival in the present study and therefore does not act as a confounding factor for other variables, nor does tumor volumetry, as demonstrated by prior findings.

In the present study, tumors presented by the patients at diagnosis were medium to large, and the extent of resection was high (median 95.9% [88.8–100]), with very low postoperative enhancing remnants. As the volumetry was highly homogeneous, no survival differences were found regarding these data. In addition, postoperative contrast-enhancing tumor volumetry showed a trend towards longer survival for smaller postoperative volumes (*p* = 0.0551), as is to be expected. Survival differences have indeed been observed for patients who completed planned adjuvant chemotherapy. A total of 59.4% of patients did not complete planned adjuvant chemotherapy, mostly due to clinical or radiological progression (except for three patients—standing for 15.8% of them—who were forced to stop adjuvant chemotherapy because of temozolomide side effects). Even though no differences were seen in PFS for patients who did or did not complete chemotherapy, most patients do not complete planned adjuvant chemotherapy because they progress intra-treatment, which does not allow them to continue with the proposed treatment. Finally, survival differences also exist for patients who present with a worse initial performance status. Having an initial ECOG >0 increases the risk of exitus almost three times (HR = 2.992) compared to patients who are initially fully active and able to carry out all pre-disease performance without restriction. Interestingly, although significance was not reached in the present study, a trend towards better survival for those patients without CDK4 mutation was also observed, aligning with the previous literature [11].

Returning to the topic of NGS, the turnaround time for NGS studies could pose a significant challenge in clinical decision-making for patient management. Therefore, the incorporation of automated NGS systems, such as the Genexus sequencer, which delivers results in less than 24 h [12], is particularly compelling, as is the use of panels like the OCA panel employed in this study, which covers a broad spectrum of diagnostic markers and potential therapeutic targets [13].

Sahm et al. [4] developed a customized NGS gene panel (intronic and promoter regions of 130 genes recurrently altered in brain tumors) and used it in 150 samples (79 retrospective cases and 71 prospective cases). They found 98% concordance compared to established, single biomarker methods, but discrepancies in TERT promoter mutation (not called by NGS) and ARTX mutations not detected by Sanger sequencing [4]. They state that although a panel containing less than 10 genes (IDH1/2, H3F3A, 1p/19q, ATRX, TP53, EGFR, NF1, BRAF, and PTEN) would resolve the majority of diagnoses on diffuse glioma samples, covering a larger set of genes and structural events can assess a larger variety of entities and identify potential targets without a significant increase in time or costs [4]. In both series, NGS has been effective in samples with low tumor cell content, and information from NGS has identified potential targets for experimental therapy: in 79% of glioblastomas in their series and 93.7% in ours.

Regarding diagnosis, prior research has identified distinct subtypes of glioblastoma —Classical, Proneural, Mesenchymal, and Neural—based on gene expression profiling. Aberrations and gene expression of EGFR, NF1, and PDGFRA/IDH1 each define the Classical, Mesenchymal, and Proneural subtypes, respectively [14]. In any case, recent developments highlight the vital role of DNA methylation profiling in enhancing glioblastoma subtyping, as highlighted in the recently published cIMPACT-NOW Update 9 [15], for methylation patterns serve as a more stable and clinically relevant marker than gene expression.

In addition to the diagnostic value, NGS not only may detect amenable targets to small molecule inhibitors and biologicals, but it also may disclose tumor-specific immune targets [4,16,17]. The Cancer Genome Atlas Research Network (TCGA) detected therapeutic targets in up to 90% of the samples of 500 primary gliomas [18]. Although we have not been able to assess treatment decision impact, experimental therapeutic targets have also been detected in the majority of the patients, as previously stated. Other studies also prove the utility of NGS paneling in the detection of actionable targets and even in leading to a treatment change.

For example, in a retrospective study of 557 patients with IDH-wt GB, NGS results were used for clinical decision-making in 23% of patients, mostly as a prerequisite for clinical trial enrolment in recurrent GB. Specific mutations were required for study inclusion in only 8% of these patients, and a survival benefit was not observed in patients enrolled in clinical trials [5].

On the other hand, Blumenthal et al. profiled 43 patients (34 GB, 8 anaplastic astrocytomas, 1 anaplastic oligodendroglioma) with a 30% treatment decision impact. Their median number of GAs per patient was 4.5 (range 1–23), comparable to ours (median number of 4, range 0–11). In their series, treatment with targeted agents included everolimus, erlotinib, afatinib, palbociclib, trametinib, and BGJ398, but none of the patients showed response to respective biologic treatments (targeting various genomic findings, including EGFR alterations, mTOR—mammalian target of rapamycin—activation, cell cycle targets, and FGFR1—fibroblast growth factor receptor 1-mutations [3].

Furthermore, a similar pattern is observed not only in glioblastoma but in other solid tumors. In a prospective study that enrolled more than 4500 patients with solid tumors for NGS panel testing (The Ontario-wide Cancer Targeted Nucleic Acid Evaluation (OCTANE) study) [13], oncologists classified as actionable the testing results of 47.7% of the patients, and in 15.7% of them, NGS results led to a change in drug treatment: 60% were enrolled in a clinical trial, 21% received an approved drug, 13% avoided ineffective treatment, and 6% were prescribed off-label therapy [13]. Despite the valuable information provided by NGS, in this study, there was no impact on the overall survival between patients who received matched treatment versus those who did not (*p* = 0.55, median survival not reached) [13].

Up to the present, evidence seems to announce that in glioblastoma, the use of NGS provides useful information for diagnosis, especially in young patients, but it does not seem to translate into a change in the overall survival of patients, even if for some of them NGS data have resulted in a change in treatment or even an enrolment in clinical trials.

However, we firmly believe this does not mean that performing NGS in young patients is not worth it. Research is the only way to progress in this field, and patients can benefit from all the information NGS brings out, provided that it is not to the detriment of treatment standards or to the detriment of patients’ quality of life. With molecularly guided treatments for some entities becoming available or being studied, high-throughput analyses have become a requirement to identify patient (mostly children and younger adults) candidates for specific treatment options.

Based on multiomic studies, mutations or amplifications in vascular endothelial growth factor—VEGF—(bevacizumab) or EGFR, NTRK fusions (entrectinib or larotrectinib), CDK4/6 gene amplification (palbociclib), and BRAFV600E mutations have been identified as potential therapeutic targets in glioblastoma, among others [4,19]. Furthermore, successful treatment of gliomas harboring BRAFV600E mutation with BRAF/MEK-inhibitors—dabrafenib plus trametinib—has been reported, with objective response rates of 33% in high-grade gliomas [20,21]. The median duration of response was 31.2 months, the median PFS was 5.5 months, and the median OS was 17.6 months [21]. In the present study, NGS detected BRAFV600E mutation in one young patient, aged 44 at glioblastoma diagnosis.

EGFR amplification is the most common alteration in primary glioblastoma. In our cohort, it is present in 13 out of 32 patients (40% of cases). While EGFR pathway inhibitors might be expected to target oncogene addiction of glioblastoma, attempts to target EGFR with antibodies or small molecules have not been proven to be clinically successful at the moment [22,23,24]. Recently, studies suggest overcoming EGFR inhibitor resistance in GB by targeting co-amplified genes in order to augment the therapeutic efficiency of EGFR tyrosine kinase inhibitors. This is the case with SEC61G, which was found to be coamplified with EGFR, and highly expressed in GB. Zeng et al. published results suggesting that depletion of SEC61G promotes the infiltration and cytolytic activity of CD8+ T cells, inhibiting GB occurrence in mice [23]. Combination therapy of EGFR-amplified glioblastomas is, therefore, a possibility that would allow the treatment of a large proportion of patients, as they account for 40–60% of all glioblastoma patients.

On the other hand, studies also suggest targeting EGFR pathways given their participation in metabolic reprogramming, which is crucial for tumor growth and immune-escape mechanisms. A recent study showed that targeting the EGFR/AKT and mevalonate pathways enhanced the antitumor effect of temozolomide in glioblastoma cells and animal models [25].

EGFRvIII (eight patients in our series, 25%), namely the epidermal growth factor receptor class III variant, is a constitutively activated mutant of the wildtype tyrosine kinase, which is present in a substantial proportion of malignant gliomas and other human cancers, but completely absent from normal tissues [16]. EGFRvIII is important when talking about glioma immunotherapy. EGFRvIII is suitable as a target of vaccination, as it is the point mutation occurring in diffuse gliomas in the gene of IDH1 (R132H) [26].

Interestingly, a very recent phase 1 clinical study confirms that EGFR is a suitable immunotherapeutic target in glioblastoma. The Intraventricular CARv3-TEAM-E T Cells in Patients with Glioblastoma (INCIPIENT) study evaluates its safety in patients with recurrent or newly diagnosed glioblastoma and shows rapid and notable radiographic responses in three participants within days after a single intraventricular infusion of dual-targeting CARv3-TEAM-E T cells [27].

Less frequent alterations, such as CDK4/6, TERT, PTEN, and PIK3CA, among others, are also the subject of multiple ongoing studies, including clinical trials [4,19,28].

Finally, it is important to emphasize that NGS is essential in particular cases where rare but important alterations may be found. It is the case with NTRK fusions, very rare in gliomas (0.55–2%)—and significantly more prevalent in young patients—where immunohistochemistry cannot be used for screening. [4] In addition, NGS can provide added value to cases where finding certain allele frequencies (at least 50%) in some genes (for example, NF1, related to neurofibromatosis, p53—Li-Fraumeni, PTEN—Cowden Sd, and TSC—Tuberous Sclerosis, among others [29,30]) may be a reason for genetic testing of not only patients but also relatives.

It is worth mentioning that not all health centers have current availability for NGS testing, which has higher direct costs than implemented single assays. Nevertheless, many neuro-oncology referral centers have access to this technology due to its implementation for other malignancies (such as lung cancer or colon cancer) and are using it daily [5,31,32].

The main limitation of the present study is its retrospective nature, which does not allow us to see the actual effect on treatment decisions. Concerning NGS, one of its limitations is the requirement for material that has not been over-fixed and is suitable for molecular biology techniques. The samples must contain a minimum tumor percentage (20%) to enable analysis. On the other hand, detecting gene fusions through NGS presents challenges in probe design for obtaining amplicons, as well as in bioinformatic processing. While the use of RNA for NGS has been accepted as a solution, it has yet to achieve the required sensitivity to eliminate the possibility of false negatives. Another limitation of NGS is the difficulty in determining copy number alterations of the analyzed genes. Although bioinformatic algorithms based on NGS-generated products exist, their accuracy is still not on par with techniques such as CGH array or FISH [33]. As regards clinical trials, it should be borne in mind that they are not always available in all countries, and in some cases, there is patient refusal of them or even an inability on the part of patients to receive further treatment because of clinical deterioration. Furthermore, to date, in most of the studies of recurrent glioblastoma, molecular criteria are not included in the study design [34].

Speaking of the future, as stated in the European Association of Neuro-Oncology (EANO) guideline [35], advanced molecular diagnostics using NGS panels or methylation arrays will probably be cost-effective in the long term because they allow for the assessment of multiple individual molecular parameters required for diagnostics (WHO classification) in a single assay. Furthermore, they shorten turnaround times and reveal information that would not have been possible with a stepwise single-assay approach.

Moreover, as we previously discussed, these high-throughput analyses will provide knowledge supporting further scientific development in neuro-oncology, and specifically in the treatment of high-grade gliomas. To note, it will be important to focus on genome-driven trials in patients with glioblastoma [34].

## 5. Conclusions

Next-generation sequencing is a valuable diagnostic tool for younger patients with suspected high-grade gliomas, as it allows for the simultaneous assessment of multiple molecular parameters within a single assay and, in some cases, can provide a diagnosis even in the absence of a definitive histopathological tumor classification. This technique can be conducted on limited tumor samples, which is particularly advantageous in cases involving eloquent resections or biopsies. Its availability may even preclude the need for repeat biopsies. Despite several unresolved questions surrounding the clinical application of molecular testing in CNS tumors, NGS is likely to become increasingly important in routine neuropathology diagnostics as new therapeutic targets and treatments continue to emerge. Currently, it plays a pivotal role not only in diagnosis but also in identifying biomarkers that enable patient participation in clinical trials and contribute to scientific advancements.

## Figures and Tables

**Table 1:** Patient characteristics (n = 32). ECOG: Eastern Cooperative Oncology Group performance status; IDH: isocitrate dehydrogenase; NGS: next-generation sequencing; MGMT: O-6-methylguanine-DNA methyltransferase; RT: radiotherapy; TMZ ChT: temozolomide chemotherapy.

Patient Characteristics	
Median age at diagnosis	47.7 years [28, 65]
Gender	
Female	10 (31.2%)
Male	22 (68.8%)
Baseline ECOG	
0	21 (65.6%)
1	11 (34.4%)
Histology (assumed at diagnosis)	
Glioblastoma, IDH not mutated, grade 4	32 (100%)
Histology (after NGS data)	
Glioblastoma, IDH not mutated, grade 4	30 (93.8%)
Astrocytoma, IDH mutated, grade 4	1 (3.1%)
H3G34 mutated Diffuse Hemispheric Glioma	1 (3.1%)
MGMT status	
Methylated	12 (37.5%)
Unmethylated	20 (62.5%)
Surgical resection	
Biopsy	2 (6.2%)
Partial	8 (25%)
Subtotal	10 (31.3%)
Gross total	12 (37.5%)
No. of patients eligible for Stupp	32
No. of patients who completed adjuvant RT	32
No. of patients who completed adjuvant TMZ ChT	13
Median progression-free survival	13 months [10, 23]
Median overall survival	25 months [18, 39]

**Table 2:** Tumor volumetry data (n = 32). Normal distribution variables are described by mean and standard deviation, and those not following normality are described by median and range (Q1, Q3). IQR: interquartile range; SD: standard deviation; FLAIR: fluid-attenuated inversion recovery; EOR: extent of resection.

Volumetry Data	
Total preoperative volume (enhancing + necrosis)	Median 21.9 cc [11.2, 46.3], IQR 35.1
Enhancing preoperative volume	Median 14.3 cc [6.1, 24.1], IQR 18.0
Necrosis preoperative volume	Median 6.6 cc [2.0, 22.2], IQR 20.2
FLAIR preoperative volume	Median 53.5 cc [22.2, 92.3], IQR 70.1
Total including FLAIR preoperative volume	Mean 91.1 cc (SD 56.0)
Total postoperative volume (enhancing + necrosis)	Median 1.1 cc [0, 2.60], IQR 2.60
Enhancing postoperative volume	Median 1.1 cc [0, 2.60], IQR 2.60
Necrosis postoperative volume	Median 0 cc [0, 0], IQR 0
FLAIR postoperative volume	Mean 46.4 cc (SD 30.3)
Total including FLAIR postoperative volume	Mean 49.1 cc (SD 30.3)
EOR	Median 95.9% [88.5, 100], IQR 11.5

**Table 3:** Patient next-generation sequencing (NGS) results. CT: clinical trials; GB: glioblastoma; OS: overall survival (months). * Samples in which cytosine deamination occurred.

Patient	Age	Number of Genetic Alterations Detected	Number of Biomarkers for CA	Genetic Alterations (Bold: Biomarkers for Clinical Trials)	Diagnosis Change	Number of Clinical Trials Potentially Eligible for	OS (Months)
**1**	64	3	1	**TP53 p**.(W146 *) c.**438G > A**, TERT c.-182C > T, ATRX p.(Q2330 *) c.6988C > T	No	1	27
**2**	63	5	4	**PIK3CA p**.**(C420R) c**.**1258T > C**, **PTEN p**.(D252Afs*18) c.**754_755insCATGTACTTTGAGTTCCCTCAGCCCGTTACCTGTGTGTGGTG**, **MAP2K2 p**.**(D70N) c**.**208G > A**, **MAP2K2 p**.(Q38 *) c.**112C > T**, SMO p.(R290H) c.869G > A	No	3	27
**3**	38	9	7	**EGFR amplification**, **PTEN p**.**(R173S) c**.**517C > A**, **AKT3 amplification**, **CDK4 amplification**, **TERT c**.**-124C > T**, **PMS2 p**.**(K541fs) c**.**1620_1621insG**, **MAP2K2 p**.(Q38 *) c.**112C > T**, MDM2 amplification, MSH6 c.3647-53_3647-37delTTTTTGTTTTAATTCCT	No	15	19
**4**	34	6	2	**IDH1 p**.**(R132G) c**.**394C > G**, **PTPRZ1-MET**.**P1M2 fusion**, ERCC2 p.(Q662 *) c.1984C > T, SMO p.(V546I) c.(1606G > A), TP53 p.(S315Lfs*30) c.943delT, CDKN2A deletion	**Yes**	11	22
**5**	42	6	3	**PTEN p**.**(K125N) c**.**375A > T**, **TERT c**.**-124C > T**, **KRAS amplification**, CCND2 amplification, CREBBP p.(S1436R), SETD2 p.(W1562R) c.4684T > A	No	7	16
**6**	64	2	0	ARID1A p.(Q1334dup) c.4001_4002insGCA, ATR p.(R1201C) c.3601C > T	No	0	34
**7**	61	3	2	**EGFR amplification**, **ATM p**.(A1954Lfs*38) c.**5858_5859insTTTAC**, MSH2 p.(P385A) c.1153C > G	No	11	50
**8**	28	11	6	**ATRX p**.(H1338Qfs*11) c.**4014_4015delTA**, **TSC2 c**.**1600-3A > G**, **TSC2 c**.**482-3C > T**, **H3-3A p**.**(G35R) c**.**103G > A**, **PDGFRA amplification**, **TP53 p**.(R342 *) c.**1024C > T**, ATR c.5739-14_5739-6delinsT, KIT amplification, KDR amplification, MRE11 p.(R380H) c.1139G > A, RB1 c.1215 + 1G > A	**Yes**	4	63
**9**	53	4	3	**EGFR p**.**(A289D) c**.**866C > A**, **EGFRvIII**, **EGFR amplification**, RET p.(G748D) c.2243G > A	No	13	28
**10**	44	3 *	2	**BRAF p**.**(V600E) c**.**1799T > A**, **EGFR::SEPT14 fusion**	No	11	47
**11**	50	3	3	**EGFR amplification**, **PIK3CA p**.**(N1044K) c**.**3132T > A**, **TERT c**.**-124C > T**	No	12	45
**12**	34	3	2	**FGFR1 p**.**(N577K) c**.**1731C > A**, **CDK12 p**.**(R44W) c**.**130C > T**, PMS2 p.(K541Efs*3) c.1620_1621insG	No	4	32
**13**	54	4	4	**EGFR amplification**, **EGFR p**.**(A289V) c**.**866C > T**, **TERT c**.**-124C > T**, **CDK12 p**.**(R44Q) c**.**131G > A**	No	12	58
**14**	41	2	2	**PTEN c**.**802-2delA**, **PIK3CA amplification**	No	2	16
**15**	55	0 *	-	-	No	-	76
**16**	58	4	4	**EGFR amplification**, **EGFRvIII**, **EGFR p**.**(G598V) c**.**1793G > T**, **PTEN c**.**492 + 1G > C**	No	14	43
**17**	49	2	1	**EGFRvIII**, MDM4 amplification	No	4	12
**18**	45	7	4	**PTEN c**.**80-2_80-1delAG**, **MAP2K2 p**.(Q38 *) c.**112C > T**, **TP53 p**.(F338Lfs*6) **c**.**1014_1017delCGAG**, **TP53 p**.**(H168L) c**.**503A > T**, ARID1A c.2162-1G > A, FGF3 p.(A23T) c.67G > A, MDM4 amplification	No	3	18
**19**	51	3	3	**PTEN c**.**161_164 + 1delTAAGG**, **ATM p**.**(V1446I) c**.**4336G > A**, **TERT c**.**-124C > T**	No	3	14
**20**	48	7	5	**EGFR amplification**, **EGFRvIII**, **EGFR p**.**(A289T) c**.**865G > A**, **EGFR p**.**(A289V) c**.**866C > T**, **CDK12 p**.**(R44W) c**.**130C > T**, TERT c.-194C > T, CDKN2AB loss	No	5	35
**21**	39	3	2	**EGFRvIII**, **EGFR amplification**, TERT c.-194C > T	No	4	14
**22**	36	6	2	**EGFR amplification**, **CDK4 amplification**, CCND2 amplification, MDM2 amplification, SMO amplification, TERT c.-194C > T	No	10	15
**23**	52	7	3	**EGFR amplification**, **EGFR vIII**, **CDK4 amplificadtion**, MDM2 amplification, SMO amplification, CCND2 amplification, TERT c.-194C > T	No	13	39
**24**	48	3	2	**TERT c**.**-124C > T**, **NF2 c**.**1341-1G > T**, ARID1A p.(W337 *) c.1011G > A	No	5	55
**25**	49	5	3	**TERT c**.**-146C > T**, **FGFR3-TACC3 fusion**, **CDK4 amplification**, MDM2 amplification, MSH6 p.(R841T) c.2522G > C	No	3	17
**26**	48	5	3	**EGFR amplification**, **EGFR vIII**, **PIK3CA p**.**(M1043I) c**.**3129G > C**, **TERT c**.**-124C > T**, CDKN2AB deletion	No	17	14
**27**	51	3 *	2	**EGFR amplification**, **EGFR vIII**, CDKN2AB deletion	No	16	12
**28**	47	2	2	**TERT c**.**-146C > T**, **FGFR3-TACC3 fusion**	No	7	67
**29**	49	6	5	**EGFR amplification**, **EGFR exon 20 insertion**, **ATM p**.(Y2755Cfs*12) c.**8624_8268delATAAG**, **TERT c**.**-124C > T**, **MAP2K2 p**.(Q38 *) c.**112C > T**	No	13	20
**30**	44	4	2	**MAP2K2 p**.(Q38 *) c.**112C > T**, **TP53 p**.**(C277F) c**.**830G > T**, TP53 p.(R267W) c.799C > T, RB1 c.1960 + 1G > A	No	1	23
**31**	46	3	2	**TERT c**.**-124C > T**, **PTEN p**.(E284Rfs*14) c.**848_849insC**, MDM4 amplification	No	6	16
**32**	48	5	5	**TERT c**.**-124C > T**, **PTEN p**.**(P95S) c**.**283C > T**, **CDK4 amplification**, **TP53 p**.**(V272L) c**.**814G > T**, **MYCN amplification**	No	8	10

## Data Availability

The original contributions presented in this study are included in the article. Further inquiries can be directed to the corresponding author.

## Supplementary Materials

The following supporting information can be downloaded at: https://www.mdpi.com/article/10.3390/cancers17050744/s1, Figure S1. Histological glioblastoma diagnosis; Figure S2. Confirmation of TERT mutations by Sanger sequencing; Figure S3. Calculation of the extent of tumor resection (EOR) by volumetry of residual contrast enhancement on early postoperative Magnetic Resonance Imaging (C and D) compared to preoperative MRI (A and B); Figure S4. Overall Survival for MGMT (O-6-methylguanine-DNA methyltransferase) promoter methylation status.
