# Supplementary material for: Clinical Impact of a Next-Generation Sequencing Approach for Glioblastoma Patients
Source: Cancers (Basel). 2025 Feb 22;17(5):744. doi: 10.3390/cancers17050744 (PMC11898826; doi:10.3390/cancers17050744)
Supplement: Supplementary file 1 [file cancers-17-00744-s001.zip › Supplementary figures legends.pdf]

Supplementary material – Figure legends

Supplementary Figure S1. Histological glioblastoma diagnosis. Upper row: hematoxylin-eosin stain showing densely cellular, infiltrative glial proliferation with frequent mitoses (arrows) (A), microvascular proliferation (B), and necrosis (C). Lower row: immunohistochemistry with antibodies: negative for IDH1-R132H (D) and ATRX nuclear expression conserved (retained)(E).

Supplementary Figure S2. Confirmation of TERT mutations by Sanger sequencing. Image from Integrative Genomics Viewer showing C228 base position (red arrow) and C250 base position (blue arrow) TERT promoter in three glioblastoma DNA samples.

Supplementary Figure S3. Calculation of the extent of tumor resection (EOR) by volumetry of residual contrast enhancement on early postoperative Magnetic Resonance Imaging (C and D) compared to preoperative MRI (A and B). In this particular case, an EOR of 80.43% (partial resection) was achieved in the surgery of a patient with a lesion within the motor cortico-subcortical area. Surgery was performed using intraoperative neurophysiological monitoring. A) preoperative 3D-T1w-contrast-enhanced (3DT1CE) sagittal MRI; B) preoperative segmentation volumetry on 3DT1CE, calculated in Medtronic S8 Software; C) postoperative 3DT1CE sagittal MRI showing early postoperative changes, as well as the residual contrast-enhancing lesion, which was located in the functional motor subcortical area; D) postoperative segmentation volumetry on 3DT1CE, calculated in Medtronic S8 Software.

Supplementary Figure S4. Overall Survival for MGMT (O-6-methylguanine-DNA methyltransferase) promoter methylation status ($p=0.0516$) HR=0.778 [IC95%= (0.365, 1.659)]. Number of patients who present MGMT status: “unmethylated” = 20, “methylated” = 12.
