# Supplementary figures and images for: Clinical Impact of a Next-Generation Sequencing Approach for Glioblastoma Patients
Source: Cancers (Basel). 2025 Feb 22;17(5):744. doi: 10.3390/cancers17050744 (PMC11898826; doi:10.3390/cancers17050744)

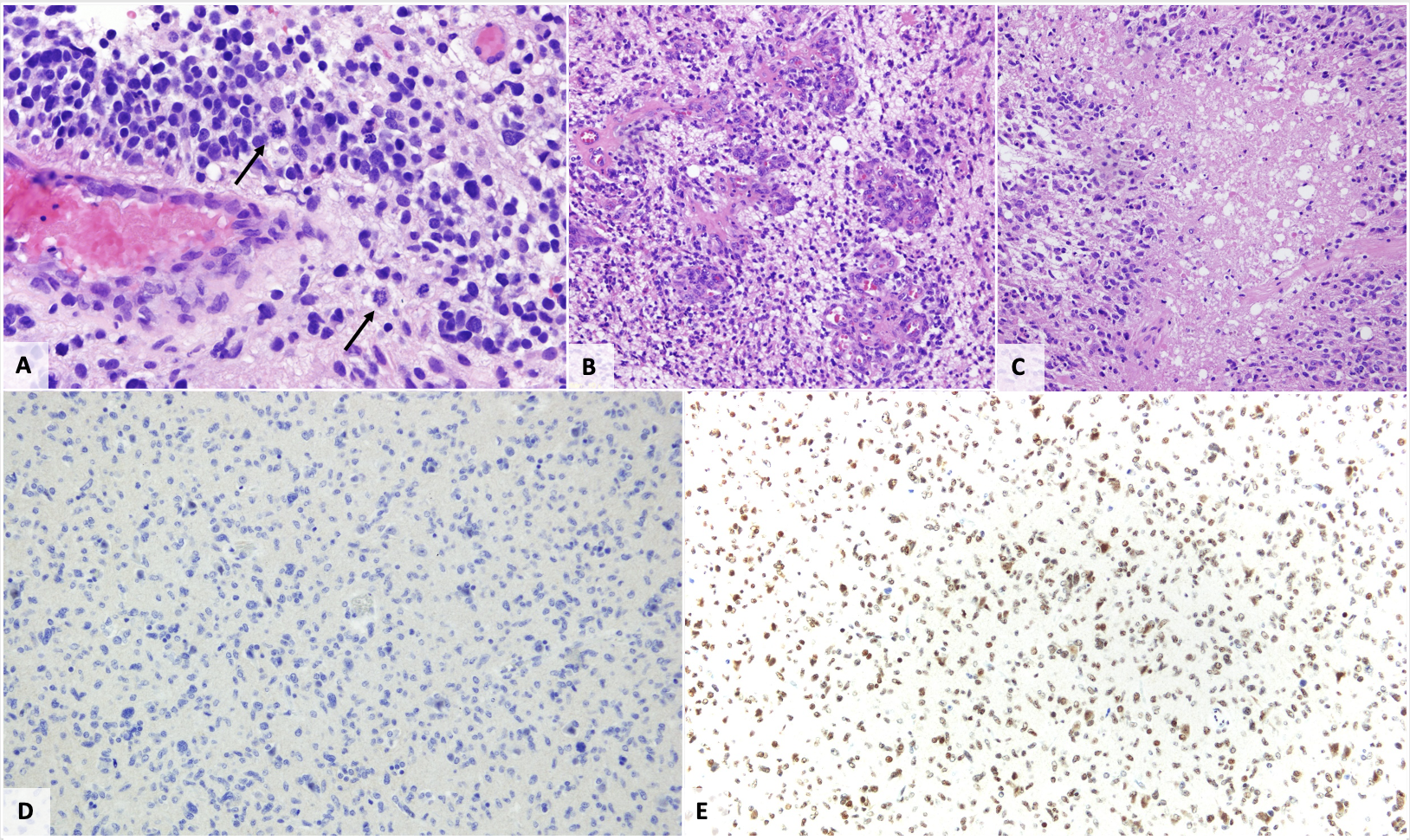

Supplement: Supplementary file 1 [file cancers-17-00744-s001.zip › Supplementary Figure S1.png]

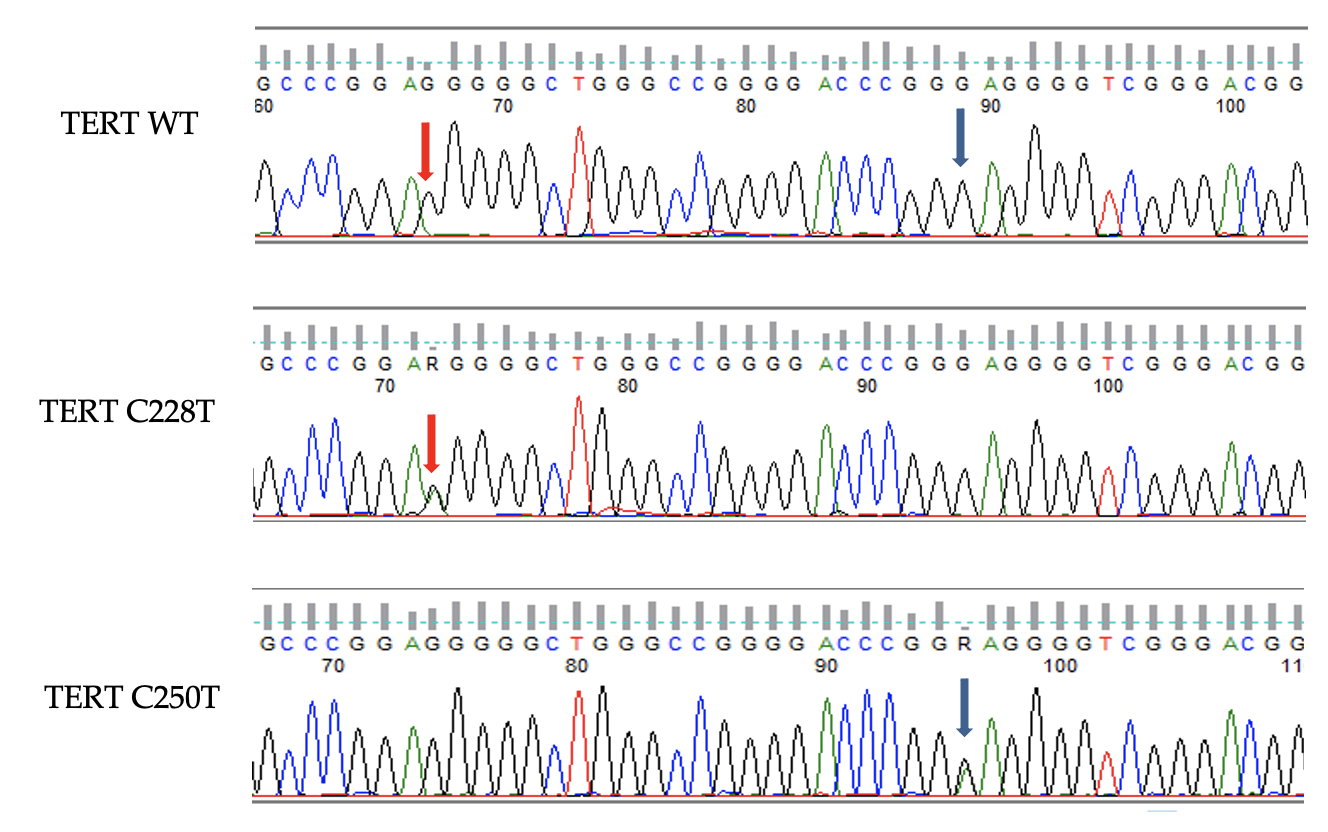

Supplement: Supplementary file 1 [file cancers-17-00744-s001.zip › Supplementary Figure S2.png]

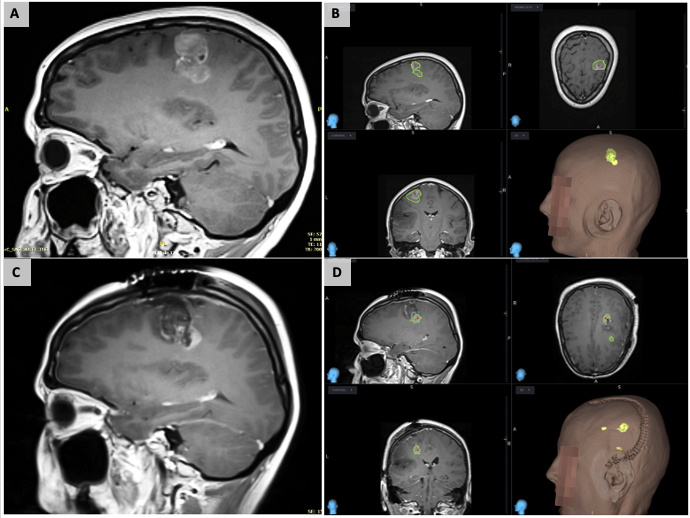

Supplement: Supplementary file 1 [file cancers-17-00744-s001.zip › Supplementary Figure S3.png]

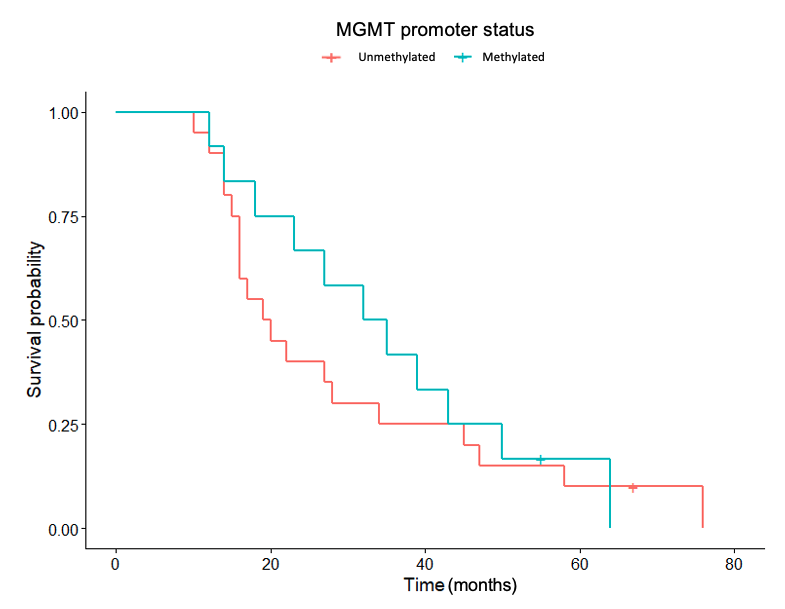

Supplement: Supplementary file 1 [file cancers-17-00744-s001.zip › Supplementary Figure S4.png]
